# A Timely Review of Cross-Kingdom Regulation of Plant-Derived MicroRNAs

**DOI:** 10.3389/fgene.2021.613197

**Published:** 2021-05-03

**Authors:** Dan Li, Jianhui Yang, Yong Yang, Jianxin Liu, Hui Li, Rongfei Li, Chunya Cao, Liping Shi, Weihua Wu, Kai He

**Affiliations:** ^1^School of Pharmaceutical Science, Hunan University of Medicine, Huaihua, China; ^2^School of Pharmacy, Hunan University of Traditional Chinese Medicine, Changsha, China; ^3^Hunan Provincial Key Laboratory of Dong Medicine, Huaihua, China

**Keywords:** microRNA, plant, cross-kingdom regulation, gene expression, activity

## Abstract

MicroRNAs (miRNAs) belong to a class of non-coding RNAs that suppress gene expression by complementary oligonucleotide binding to the sites in target messenger RNAs. Numerous studies have demonstrated that miRNAs play crucial role in virtually all cellular processes of both plants and animals, such as cell growth, cell division, differentiation, proliferation and apoptosis. The study of rice MIR168a has demonstrated for the first time that exogenous plant MIR168a influences cholesterol transport in mice by inhibiting low-density lipoprotein receptor adapter protein 1 expression. Inspired by this finding, the cross-kingdom regulation of plant-derived miRNAs has drawn a lot of attention because of its capability to provide novel therapeutic agents in the treatment of miRNA deregulation-related diseases. Notably, unlike mRNA, some plant miRNAs are robust because of their 3′ end modification, high G, C content, and the protection by microvesicles, miRNAs protein cofactors or plant ingredients. The stability of these small molecules guarantees the reliability of plant miRNAs in clinical application. Although the function of endogenous miRNAs has been widely investigated, the cross-kingdom regulation of plant-derived miRNAs is still in its infancy. Herein, this review summarizes the current knowledge regarding the anti-virus, anti-tumor, anti-inflammatory, anti-apoptosis, immune modulation, and intestinal function regulation effects of plant-derived miRNAs in mammals. It is expected that exploring the versatile role of plant-derived miRNAs may lay the foundation for further study and application of these newly recognized, non-toxic, and inexpensive plant active ingredients.

## Introduction

MicroRNAs (miRNAs) are a category of endogenous, highly conserved, 18-24 nucleotides single stranded RNAs. By binding to the 3′-untranslated region of various target genes, miRNAs play a crucial role in cell growth, cell division, differentiation, proliferation, and apoptosis (Chen et al., 2006; Guo et al., 2010). It is believed that lin-4 is the first discovered miRNA, which targets lin-14 mRNA in *Caenorhabditis elegans* (Lee et al., 1993). Thereafter, tens of thousands of miRNAs have been identified from mammals, plants and microorganisms.

Mechanisms of miRNA gene transcription and maturation were mainly revealed using *Caenorhabditis elegans* (Knight and Bass, 2001), *Drosophila melanogaster* (Hammond et al., 2000) and human cell lines (Lee et al., 2002). Although no homologs of Drosha and DGCR8/Pasha have been found in plants, RNase III Dicer, which is responsible for processing pre-miRNAs into mature miRNAs, is highly conserved in plants and animals (Kim, 2005). It is reported that the miRNA in plants and animals were processed in a similar manner (Reinhart et al., 2002). Normally, mature animal miRNAs drive from pri-miRNA, which is transcribed by RNA polymerase II/III from miRNA genes. Then these longer, hairpin loop-containing pri-miRNA is cleaved by microprocessor complex to generates about 70 nucleotides stem loop pre-miRNA. After transport from nucleus into the cytoplasm, pre-miRNA is further cleaved by RNAse III enzyme dicer and transactivation-response RNA-binding protein to produce mature miRNAs (Akbari Kordkheyli et al., 2019). Studies have revealed that miRNAs can regulate the translation of over 60% of protein-coding genes in animals (Esteller, 2011). Some miRNAs target specific gene, while, crucial miRNAs may mediate the expression of hundreds of genes simultaneously. The recent discovery and characterization of miRNAs not only add the rapidly growing repository of the pathogenic mechanisms of diseases, but also offer opportunities to applying these small molecules to therapy various disorders. Traditionally, the chemical compounds in herb or plant extracts were considered as the main active ingredients, and responsible for the observed pharmacological activities. Recently, people begin to notice that nutrients and bioactive compound, such as flavonoids, alkaloids and diterpenoids could regulate the crosstalk of miRNA, lncRNA and circRNA, and exert pro-apoptosis, anti-inflammation, anti-proliferation, anti-atherosclerosis and anti-infection activities (Bruna et al., 2017; Dong et al., 2019). Specifically, medicinal plants derived miRNAs are being recognized as a class of new bioactive compounds in nowadays. For instance, as one of the most valuable herb medicines in China, *Panax ginseng* C. A. Mey (Panax ginseng) is well known for its regulation of immune function, anti-tumor, cardiovascular protection, and cognitive improvement activities. Using Illumina HiSeq platform, Wang et al. (2019) identified 298 known and 3,500 possible novel miRNAs in *Panax ginseng*. Bioinformatics analysis showed that 2686 miRNAs in ginseng may regulate 50992 potential human genes, which involving 296 signaling pathways. Among 24 significantly enriched pathways, 3 were tightly associated with bladder cancer, small cell lung cancer, and cancer cell metabolism. Four significantly enriched pathways were related to signal transduction pathway, including Hedgehog, TGF-β, Hippo and neuroactive ligand receptor pathway. The results suggested *Panax ginseng* miRNAs are responsible, at least partially, for the clinical efficacy of *Panax ginseng*. Moreover, as a family of versatile molecular regulators, miRNAs from plant not only regulate host-gene expression, importantly, by dietary intake, exogenous plant miRNAs also affect the physiological and pathological conditions of mammals. Shahid et al. (2018) reported that while parasitizing *Arabidopsis thaliana* Heynh, *Cuscuta campestris* haustoria affects the growth and pathogenesis of *Arabidopsis thaliana* by inducing many 22 nt miRNAs, some of them could reduce mRNAs accumulation, stimulate secondary siRNA production, or promote mRNA cleavage of the host. Zhang et al. (2012) have demonstrated for the first time that through food intake, exogenous plant MIR168a can be detected in the sera and tissues of animals, and served as signaling molecules in intercellular communication. After feeding mice with total RNA extracted from fresh rice, or synthetic MIR168a, or synthetic methylated MIR168a, the levels of MIR168a in mouse serum and liver were all elevated 3 h after feeding. By binding at nucleotide sequence CCAAGCG, located on the open reading frame of low-density lipoprotein receptor adapter protein 1 (LDLRAP1), MIR168a inhibits LDLRAP1 expression, and thereby decreases LDL removal from mouse plasma. This pioneering work indicating that ingestion of plants containing high-content of functional miRNAs might be a promising way for clinical applications, because deregulation of miRNAs is involved in the pathogenesis of many human diseases such as cancers, immunodeficiency, cardiovascular diseases, and, with the new data emerging, lung diseases (Boateng and Krauss-Etschmann, 2020). More importantly, rapidly accumulating data have implicated both endogenous and dietary exogenous miRNAs regulate gene expression mainly at the post-transcriptional level via direct cleavage of transcripts (Sanchita et al., 2018; Ren et al., 2019). The high stability of plant miRNAs lays the foundation for their therapeutic application and these exogenous can survive in animals and human gastrointestinal tract (Liang et al., 2014). For instance, after drank watermelon juice, 10 miRNAs can be detected at high basal levels in human plasma among the selected 16 miRNAs (Liang et al., 2015). Unlike mRNA, plant miRNAs are robust during homogenization, mammalian circulation and even boiling processes for the following reasons (Xie et al., 2016): (1) some plant miRNAs are 2′-O-methylated on their 3′ end, this modification has a protective effect on miRNA stability. (2) The primary sequence (like high G,C content), structure of miRNA and absence of RNases digestion motifs also contribute to the stability of miRNAs (Yang et al., 2018). (3) the miRNAs protein cofactors like high-density lipoproteins (Vickers et al., 2014), and Argonaute proteins prevent them from decay. (4) herb ingredients of plant metabolites not only create a favorable environment for miRNAs but also exert RNase inhibitory activities (Xie and Melzig, 2018; Vora et al., 2019). (5) During transportation, plant miRNAs are packaged into microvesicles or exosomes, which protect them against degradation. Moreover, the plant miRNA-like miRNAs, mimicked from mammalian tumor suppressor miRNAs by methylation at 2′position of the ribose of the 3′terminal nucleotide, effectively reduced tumor burden in ApcMin/ + mice of colon cancer model (Mlotshwa et al., 2015). This observation has led to the idea that apart from plant derived therapeutic miRNAs, functional miRNAs could be produced by edible plants using bioengineering to meet the increasing demand of therapeutic miRNAs in future.

**FIGURE 1:**
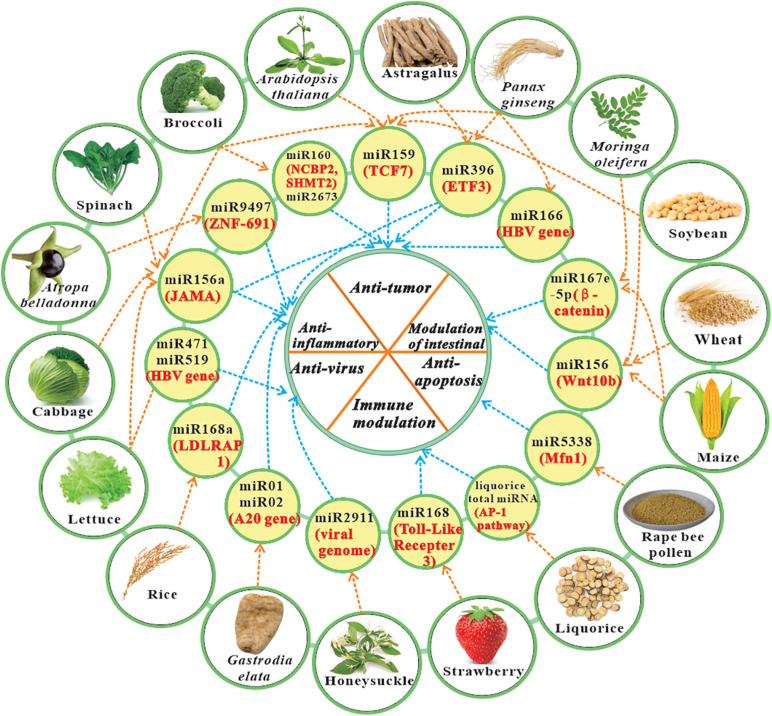
The representative plant-derived miRNAs with cross-kingdom bioactivity and their role in cross-kingdom communications. Note: The information in yellow circle contains miRNAs derived from specific plants (black text) and their main targets (red text). Some conservative miRNA family can be found in many different plant species. Other information like miRNA sequence and references can be found in Table 1. ETF3, Endothelial transcription factor 3; HBV, Hepatitis B virus; JAMA, Junctional adhesion molecule A; LDLRAP 1, Low-density lipoprotein receptor adapter protein 1; Mfn1, Mitochondrial fusion requires fusion protein 1; TCF7, Transcription factor 7; ZNF-691, zinc-finger transcription factor 691.

In 2018, Wang et al. (2018) have reviewed several studies concerning plant miRNAs regulate gene expression in animals (mainly honey bee), and the effect of hosts miRNAs on pathogens. Zhao et al. (2018) have highlighted some discoveries of both intra- and inter-kingdom communication of plant and animal miRNAs. Because current study for plant miRNAs is advancing rapidly, it is necessary to provide timely updates. This paper summarizes the plant-derived miRNAs with cross-kingdom activity in mammals (Table 1), and reviews the updated evidences regarding potential therapeutic benefit of miRNA cross-kingdom activity, like anti-virus, anti-cancer, anti-inflammation, and immune modulation, etc. (Figure 1). The current review may facilitate the recognition of a more profound understanding of the therapeutic value of plant miRNAs, and the application of these new natural resources.

**TABLE 1:** Detailed information on plant-derived miRNAs with cross-kingdom activity in mammals.

**No**	**Name of miRNA**	**Original plant**	**MiRNA sequence**	**MiRNA Target**	**References**
1	miR168a	Rice	UCGCUUGGUGCAGAUCGGGAC	Human/mouse low-density lipoprotein receptor adapter protein 1	Zhang et al., 2012
2	miR2911	Honeysuckle	AGGGUCGGGCAGGGGGCCGG	The PB2 or NS1 gene of viral genome of influenza A virus (H1N1, H5N1, H7N9)	Zhou et al., 2015
3	miR2911	Honeysuckle	AGGGUCGGGCAGGGGGCCGG	The viral genome of the novel Coronavirus SARS -CoV-2	Zhou et al., 2020
4	miR471	Lettuce	UCCCAUAGGAAUCUUGUGAAA	Hepatitis B virus (HBV)	Zhang et al., 2019
5	miR519	Lettuce	ACAAAUGGCACUAGUAAACUG	Hepatitis B virus (HBV)	Zhang et al., 2019
6	miR159	Glycine max *Arabidopsis thaliana*	UUUGGAUUGAAGGGAGCUC	Transcription factor 7 of breast cancer cell	Chin et al., 2016
7	gma-miR159a-3p	Soybean	UUUGGAUUGAAGGGAGCUCUA	Transcription factor 7 of human colonic Caco-2 cancer cell	Liu et al., 2020
8	MiR171 variant	*Arabidopsis*, tomato, etc.	UGAUUGAGCCGCGCCAAUAUC	mTOR pathway of HEK293 cell	Gismondi et al., 2021
9	FvmiR168	Strawberry fruits	UCGCUUGGUGCAGGUCGGGAA	Toll-Like Receptor 3 of T cell and mouse	Cavalieri et al., 2016
10	miR-396	Astragalus	UUCCACAGCUUUCUUGAACUU	Endothelial transcription factor 3 of Th2 cells in mouse	Shen et al., 2019; Abla et al., 2019
11	Gas-miR01	*Gastrodia elata*	UUCAAUAAAGCUGUGGGAAA	A20 gene of 293T cell and mouse	Zhou, 2018
12	Gas-miR02	*Gastrodia elata*	GUUCAGGAAUGCUGUGGGAAG	A20 gene of 293T cell and mouse	Zhou, 2018; Xia et al., 2020
13	miR156a	Cabbage, Spinach and Lettuce	UGACAGAAGAGAGUGAGCAC	Junctional adhesion molecule A of human endothelial cell	Hou et al., 2018
14	aba miRNA 9497	*Atropa belladonna*	UUUCUGAAUCCAUUGUUUAUC	zinc-finger transcription factor ZNF-691 of HNG cells	Avsar et al., 2020
15	miR5338	Rape bee pollen	UGAAGCUUCAGUUGGUUGUAU	Mitochondrial fusion requires fusion protein 1 in rat	Chen et al., 2018; Peng et al., 2011
16	miR167e-5p	*Moringa Oleifera* and Maize	UGAAGCUGCCAGCAUGAUCUG	β-catenin of IPEC-J2 and Caco-2 cell	Li et al., 2019a
17	miR156	Soybean, Wheat and Maize	UGACAGAAGAGAGUGAGCAC	Wnt10b in mouse	Li et al., 2019b
18	mdo-miR7267-3p	Ginger	CAUCCAGCCAUCCACCCCAGG CCAUC	LGG monooxygenase ycnE of mouse gut microbiota	Teng et al., 2018

## Anti-Virus

The relationship between miRNAs and viral replication is complex and not completely understood. Some miRNAs could target viral genomes and proteins to suppress virus replication (Wen et al., 2013). While, other miRNAs, like gga-miR-142-5p (Ouyang et al., 2018), miRNA-16-5p (Duan et al., 2020), may inhibit host defense and enhance virus replication and infection (Li and Zheng, 2020). There is evidence that miRNAs exert antiviral effects on virus infection by modulation of certain immune and antiviral pathways, such as the janus kinase-signal transducer and activator of transcription (JAK-STAT), toll-like receptor, and mitogen-activated protein kinase signaling pathways (Lin et al., 2016; Fu et al., 2018).

Facing the novel Coronavirus SARS-CoV-2 pandemic, Chauhan et al. (2020) have indicated that using miRNAs that can binding to spike protein or the genome of SARS-CoV 2 is a promising way to fight back against this viral disease. *Lonicera japonica* Thunb (honeysuckle, HS)-encoded atypical miRNA2911 is considered as the first active plant miRNA identified in Traditional Chinese Medicine (TCM), which inhibiting the replication of influenza A viruses, including H1N1, H5N1 and H7N9. Three days gavage of HS decoction significantly elevated the MIR2911 level in mouse peripheral blood and lung. Besides, synthetic MIR2911 gavage administration reduced the viral infection-induced weight loss and even mortality in mouse. The antiviral activity of MIR2911 may attributed to its ability to bind to the nucleotide sequences of H1N1 virus-encoded PB2 and NS1 genes (Zhou et al., 2015). The authors further demonstrated that MIR2911 in honeysuckle decoction (HD) had 28 binding sites in the SARSCoV-2 genome and was able to inhibit virus replication and accelerate the negative conversion of infected patients. *In vitro*, exosomal-MIR2911 inhibited 93% of viral replication. Patients who intake 30 g dried honeysuckle (MIR 2911 level: 10.5 pmol) per day for 7 days exhibited a dramatically higher negative conversion rate compared with TCM mixture control group. Besides, HD-MIR2911 also reduced the time taken to become SARS-CoV-2 PCR-negative in both male and female patients (Zhou et al., 2020). Zhang et al. (2019) generated two plant-derived small silencing RNAs (amiR471 and amiR519) in edible *Lactuca sativa* L. var. ramosa Hort (lettuce) and found that these miRNAs could inhibit HBsAg expression. Using HBsAg^–/+^ transgenic mice, the authors further revealed that amiR519 lettuce decoction decreased both the fat droplets and immune-associated factors, such as TNF-α, IL-6, CCL5 and IFN-γ in mice liver. Long-term feeding with amiRNA-containing decoction showed no toxicological effects but alleviated liver injury in HBsAg^–/+^ transgenic mice caused by persistent HBsAg expression. Using bioinformatics approach, Perez-Quintero et al. (2010) have shown that plant miRNAs, including miRNA families of 156, 395, 159, 166, and 160 can target the coding regions of RNA polymerases, cylindrical inclusion proteins, capsid proteins, and nuclear inclusion body proteins in plant-infecting viruses. Due to a higher matching percentage for plants miRNAs to plant viruses than animal and other source of miRNAs, plant miRNAs target plant-infecting viruses more efficiently. Similarly, the following study on screening conserved plant miRNAs against the hepatitis B and C viruses showed that five plant conserved miRNAs: miR-166, 169, 172, 390 and 399 were quite likely to bind to the six HBV gene sequences. While, miR-156/157, 166, 169, 172 and 390 were predicted to target the six HCV gene sequences (Barozai and Din, 2017).

## Anti-Tumor

The anti-tumor effects of plant-derived miRNAs were mainly achieved by down-regulating the critical targets or pathways in tumorigenesis, and exerting tumor-suppressor function (like miRNA34). By sequence analysis, four common miRNAs in edible plants were found to be perfect match with 22 human transcripts. These plant miRNAs targeted genes are involved in muscle contraction and tumor suppression, indicating that food miRNAs may altering our body functions by regulating human genome (Kashani et al., 2019). Recently, microvesicles purified from *Moringa oleifera* seeds increased apoptosis levels of Jurkat and Hela cells. BCL2 protein expression and mitochondrial membrane potential of the two cell lines were also significantly downregulated after treatment (Potestà et al., 2020). The extracts of *Camptotheca acuminata* Decne (*C. acuminate*) have been used for thousands of years in China and Indian for the treatment of various cancers. Thirty-three stable novel miRNAs in *C. acuminate* were found to regulate 152 target genes in human. These predicted target genes are significantly associated with vital cell functions like focal adhesion, lipolysis in adipocytes and mTOR pathways. Thus, miRNAs in *C. acuminate* have great potential in modulation of cancer pathways (Kumar et al., 2017). Pirrò et al. (Stefano et al., 2016) have identified 94 conserved and 2 novel miRNAs in *Moringa oleifera*. Bioinformatics prediction have revealed that these miRNAs may regulate apoptosis, cell cycle and protein degradation related human genes. Additionally, transfection of *Moringa oleifera* mol-mir168a to HepG2 cells significantly decreased SIRT1 protein level, suggesting a key role of mol-mir168a in modulation of cell cycle, apoptosis, and inflammation in human. By profiling small RNAs in the sera of 42 breast cancer patients, Chin et al. (2016) found that the serum level of plant miR159 (present in *Arabidopsis thaliana* Heynh, *Glycine max* Merr and *Brassica oleracea* L. var. capitata) is inversely correlated with breast cancer incidence and progression. Oral intake of miR159 at the concentration of 25 mg/kg for 16 days dramatically reduced tumor growth in tumor xenograft models. In addition, the expression of transcription factor 7 (TCF7) and potent oncogene MYC were significantly down-regulated. Mechanism study revealed that synthetic miR159 had the potential to inhibit the growth of breast cancer cells by aiming the region of nucleotide 1445-1470 and 1842-1870 in 3′UTR of TCF7, then resulting in the decrease of MYC protein levels. Similarly, *Glycine max* Merr (soybean)-derived Gma-miR159a, accounts for 83.3% soybean miRNA families, was also reported to promote the apoptosis of Caco-2 cells by inhibiting TCF7 expression (Liu et al., 2020). A controlled intervention study involving 19 volunteers showed that two weeks administration of 80 g *Brassica oleracea* L (broccoli) significantly increased the serum level of miR160, miR2673 in a dose and time-dependent manner. Transfection of miR160 mimic significantly downregulated CDC6, POL2RF, PYCR1, MTHFD2, NCBP2, SHMT2; CELSR3 and FKBP4 expression in cancer cell lines. Among them, NCBP2 and SHMT2 were significantly reduced at mRNA and protein level. Thus, except for the chemical compounds, *Brassica* miRNAs may also attribute to its cancer prevention function (Pastrello et al., 2016). Junctional adhesion molecule A (JAMA) is a transmembrane protein and plays crucial role in cell invasion, platelet aggregation and lymphocyte adhesion. Previous research has identified 84 conserved miRNAs and 184 novel miRNAs from broccoli, and noticed that miR156a is the most highly expressed miRNA in flower and leaf (Tian, 2014). By binding to the JAMA mRNA 3′-UTR region, miR156a inhibited JAMA protein expression and result to down-regulation of Vimentin, up-regulation of E-cadherin in human nasopharyngeal carcinoma cell lines CNE2 and HONE1. These effects were also accompanied by a remarkable reduction of invasion ability of CNE2 and HONE1 cell. MiRNA171 is a ubiquitous and conserved plant miRNA. Recently, it is shown that miRNA171 significantly decreased G protein subunit alpha 12 mRNA and protein levels of human embryonic kidney 293 cells, and then modulated mTOR pathway, one of downstream signaling factors of GNA12. Thus, miRNA171 may be of great value in the treatment of GNA12 over-expressed carcinomas (Gismondi et al., 2021).

The anti-tumor effect of human miRNA34 families is different from the above-mentioned miRNAs. Human miRNA34 families are well known as tumor-suppressor miRNAs, play an important role in apoptotic cellular mechanisms by repressing BCL2 mRNA (Dang et al., 2013). A recent study showed that *Olea europaea* Linn miRNAs, oeu-sR20, oeu-sR27 and oeu-sR34 are homologous to human miRNA34 in cross-kingdom regulation of tumorigenesis. Like hsa-miR34a, transfect *Olea europaea* miRNAs mimics to THP1 and Jurkat cells affects cell viability and apoptosis via significantly decrease SIRT1 and BCL2 proteins expression (Minutolo et al., 2018). These results indicate *Olea europaea* miRNAs have great potential to be used as a novel, natural non-toxic, anticancer drug.

## Immune Modulation

MicroRNAs have emerged as an important regulator in the mammalian immune system, as they participate in various critical events, such as development, homeostasis, and regulation of multiple pathways. For instance, *Glycyrrhiza uralensis* Fisch (Liquorice) miRNA induced obvious proliferation and aggregation of human peripheral blood mononuclear cells (PBMC). Moreover, the proportion of HLA-DR^+^ cells was significantly increased from 16.7% to 68.1%, which indicating that liquorice miRNAs improve immune function by stimulate PBMC growth (Shao et al., 2015). Later study further demonstrated that immune modulation activity of glycyrrhiza miRNA was related to its effect on the regulation of genes involved in T cell differentiation, inflammation and apoptosis. Transfection of glycyrrhiza miRNA to PBMC inhibited Th2 differentiation by inhibiting AP-1 signaling pathway. Besides, the down-regulated NF-κB, Bax and p53 expression proves its anti-inflammation and anti-apoptosis activity (Xiang et al., 2017). Cavalieri et al. (2016) have suggested that dietary plant miRNAs may reduce chronic diseases by regulating inflammation response. FvmiR168 interacts with dendritic cells (DCs) by binding to the ectodomain of TLR3, leading to the reduction of cytokines release and inhibition of T cell proliferation. Injection of “cocktail” plant sRNA to experimental autoimmune encephalomyelitis (EAE) mice significantly reduced disease onset and maximum EAE score. Besides, plant sRNA-treated mice had less CD11c infiltrates in spinal cord areas. These were attributed to the reduction of serious inflammatory cytokines by plant sRNA via down-regulating the expression of master regulators of pro-inflammatory including Tbet, Rorc and IL-10 master regulator FoxP3. JAK-STAT pathway regulates cell growth, proliferation, differentiation, and apoptosis, and thus critical to immune and inflammatory responses (Jin et al., 2018). By analysis of 410 human plasma small RNA sequencing data, Liu et al. (2017) have found that high relative amount of plant miRNA peu-MIR2910, which conserved in maize, melon, sorghum, *Solanum lycopersicum* Mill (tomato), and tea, can be detected in the plasma samples. Moreover, two seed sequences of miR2910 are the same as human miR-4715-5p and miR-4259. The authors further explored that miR2910 may target on 5’UTR of LIMA1, and the CDS of a group of genes like SPRY4, CTNND1, LBX1, STK38, etc. Since SPRY4 is involved in JAK-STAT signaling pathway, it is possible that plant miR2910 can be applied for the treatment of various diseases resulted from deregulation of the JAK-STAT pathway.

## Anti-Inflammation

MicroRNAs exert significant impact on inflammatory process by targeting proteins that response for transmit of intracellular signals, and by reducing mRNAs that translate cytokines. Sharma et al. (2017) have identified thirty-four mature *Curcuma longa* L. Among them, clo-mir-14 showed sequence homology with human miRNA-4693-5p. A total of twenty human genes such as regulator of G-protein signaling 13, adenylate cyclase 9, gap junction protein A1, calcitonin receptor, etc. were predicted as target of clo-mir-14, indicating that clo-mir-14 may evoke the cellular immune response during inflammation of Rheumatoid Arthritis. By intranasal delivery, *Astragalus membranaceus* Moench (Astragalus)-derived miR-396 showed about ten times higher expression in the spleen, peripheral blood and lung tissue of asthmatic mice compared with internal reference gene. After seven times consecutive treatment with 20 μg miR-396 mimics, the levels of IL-5, IL-13 cytokines were significantly reduced compared to asthmatic group, moreover, the expression of the master transcription factor GATA-3 of Th2 cells was also down-regulated by miR-396 treatment (Shen et al., 2019). As a precious Chinese herbal medicine, *Gastrodia elata* Bl (GE) possesses anti-convulsant, neuroprotection, anti-inflammatory and immune regulation effects. By Illumina high-throughput sequencing, 5718 miRNAs were discovered from GE, of which 52 were new GE miRNAs. Function prediction showed that Gas-mi R01 and Gas-mi R02 might interfere with three key genes: A20, TNF-α and IκBα. Transfect Gas-mi R01 and Gas-mi R02 mimics to 293T cell significantly inhibited the expression of A20, which is an important feedback regulator of NF-kB-driven gene. Additionally, after gavage GE decoction to mice, high levels of Gas-mi R01 and Gas-mi R02 could be detected in mice serum, liver, brain, kidney, spleen, and reduced A20 expression (Zhou, 2018; Xia et al., 2020). These results suggested that GE miRNAs are a group of active ingredients in GE and may responsible for the well know anti-inflammatory and immune regulation efficiency of *Gastrodia elata.* Except for the cholesterol metabolism modulation role of MIR168a (Zhang et al., 2012), MIR156a was also reported to exert beneficial effects on cardiovascular disease (CVD). In a controlled intervention study, after eating 500 g lettuce, miR156a was stably presents in human serum and reached its peak value one to three hours after ingestion. Moreover, CVD patients have exhibited a decreased MIR156a level in serum and blood vessel. Plant MIR156a combines with 3′-UTR of JAMA mRNA to suppress the junction JAMA expression and mediate the cleavage of JAMA mRNA, thus inhibiting inflammatory-induced monocytes adhesion in CVD patients (Hou et al., 2018). Aba miRNA 9497 derived from Atropa belladonna *L*, has a highly homologous sequence to miRNA-378 of *Homo sapiens*. Avsar et al. (2020) have demonstrated for the first time that both aba miRNA 9497 and miRNA-378 could downregulate the mRNA expression of human CNS-enriched ZNF-691 by binding to 3′-UTR of this gene. The authors speculated that the neurotoxic actions of Atropa belladonna may partially results from the modulation role of ZNF-691 by aba-miRNA-9497. Since ZNF-691 has been involved in many neuropathological roles such as inflammation, targeting ZNF-691 by aba miRNA 9497 should be of great interest as the crucial role of inflammation in various diseases.

## Anti-Apoptosis

Apoptosis is a cell death process occurring when cells face irreversible stress, and its deregulation in humans will result in various proliferative and degenerative diseases. Normally, proteins of anti-apoptotic family members (BCL2, MCL1) are binding to Beclin 1, an important autophagy-promoting member, to maintain cellular homeostasis. Cell stress can cause the disassociation of these two kinds of proteins, thus promoting autophagy and inhibiting apoptosis, respectively. Many miRNAs regulate autophagy and apoptosis signals by modulating the above-mentioned protein-protein interactions (Xu et al., 2012). Using testosterone propionate induced benign prostatic hyperplasia (BPH) rat model, Chen et al. (2018) have shown that miRNA derived from the pollen of *Brassica napus* L (rape bee) could cross the blood-prostate barrier and entry prostate. After gavage fed rape bee pollen to BPH rats at the concentration of 6.39 g/kg for 3 weeks, the abundance of seven plant miRNAs such as miR894, miR5338, miR3440-5p, miR2878-5p, miR7754-5p, miR5015, and iR7731-3p were elevated in the prostate gland of rats, accompanied by a decreased posterior lobes index compared with the model group. Further study has revealed that the most enriched miRNA in rat’s prostate by rape bee pollen treatment is miR5338, which prevents BPH by decrease Mfn1 expression and inhibit mitochondrial fusion, cell apoptosis via Bcl-2 pathway.

## Modulation of Intestinal Function

The intestine is not only essential for digestion and absorption, but also crucial for secretion and immunity, as well as mediating various bile acids or microbiota involved signaling pathways. Recent studies have revealed that plant MIR167e-5p elicits cross-kingdom effects on the proliferation of enterocyte. MIR167e-5p, a conserved and highly expressed miRNA in *Moringa oleifera* Lam and *Zea mays* L (maize), significantly reduced IPEC-J2 and Caco-2 cell proliferation in a dose-and time-dependent manner. This event was achieved by downregulating the mRNA and protein levels of β-catenin, which is a crucial target in intestinal epithelium development (Li et al., 2019a). The same group further demonstrated that MIR156, a common plant miRNA can be found in soybean, *Triticum aestivum* L (wheat) and maize, regulates intestinal development and health *in vitro* and *in vivo*. MIR156 may bind to the 3′-UTR sequence of Wnt10b, then suppresses the proliferation of IPEC-J2 cells. In mice study, MIR156-containing maize diet administration significantly reduced the villus height:crypt depth ratio in mice duodenum, accompanied with down-regulated expression of Wnt10b and β-catenin. Additionally, 7 days treatment of 300 pmol synthetic MIR156 significantly decreased the mRNA levels of Cdx2, Wnt10b, β-catenin and cyclin D1 in mice intestine (Li et al., 2019b). These studies provide evidences that plant miRNA156 and miRNA167e-5p could regulate Wnt/β-catenin pathway to maintain intestinal epithelium homeostasis and prevent colitis.

## Discussion

The discovery of miRNAs and plant miRNA cross-kingdom regulation in *Homo sapiens* has generated considerable excitement. According to the latest information in miRNA database 22, there are 38589 mature miRNAs from nearly 300 species (miRBase 22 release, October, 2018). Plant miRNAs are crucial for many aspects of plant biology, such as leaf morphogenesis, flowering time, stress response and signal transduction. Zhang H. et al. (2016) have investigated the target genes of 25 miRNAs in *Arabidopsis thaliana* in both human and *Arabidopsis*. The results showed that *Arabidopsis* miRNAs may exert similar functions such as stress response and ion transport in both human and plant, suggesting a key role of exogenous plant miRNA on physiological function of human body. According to the sequence similarity, miRNAs can be grouped into different families. In *Solanum tuberosum* L (potato), 982 miRNAs have been classified in 71 miRNA families, and as the largest family, miR1128 family have 141 members (Mian, 2014). The specific miRNA can modulate multiple gene targets, for instance, miR168-5p, one variants of the plant miRNA168a, is wide distributed in 28 plants species of 15 plants families and has the potential to regulate 123 genes of human transcriptome (Javed et al., 2017).

Plant miRNAs seem to be a promising alternative for further preclinical use, due to their diverse expression patterns and the ability to regulate many development and physiology process. Besides, widespread deregulation of miRNA expression can be found in many human diseases (Lina et al., 2020; Mansoor et al., 2020). Strategies to target miRNAs in human disease involving two aspects, one is to inhibit miRNA function use antisense oligonucleotides or “miRNA sponges.” The other is restoring miRNA function by epigenetic drugs, miRNA virus delivery system or ingestion plants/herb medicine that rich in specific miRNA (Esteller, 2011). Thus, using abundant, easy to obtain exogenous miRNA would be a promising choice and new alternative way for human diseases treatment. Plant derived nanoparticles which mainly contains miRNA, lipids, and proteins have been demonstrated to function as natural therapeutical agents for cancer, colitis, and inflammatory diseases (Zhang M. et al., 2016). Lukasik et al. (2018) reported that five plant food-derived miRNAs, including miR156a, miR166a, miR157a, miR168a, and miR172a can be found in breast milk samples of healthy volunteers. The bioinformatics results indicated that these plant miRNAs may interact with targets involving protein transport, immune processes, and signal transduction, etc. in faints. As for the regulating mechanisms, accumulating evidences demonstrated that plant derived miRNAs exert their activities both directly and indirectly. For instance, in plants and animals, miRNAs regulate gene expression by recognizing a complementary sequence, which is located in 3′UTR, ORF (Zhang et al., 2018), or 5′UTR of the targeted mRNA, with the assistance of RNA-induced silencing complex. This combination generally results in translation suppression or mRNA decapping and 5′-to-3′ decay. Besides, most plants and few animal miRNAs are highly complementary to their target mRNA, leading to endonucleolytic cleavage of their mRNA targets (Ameres and Zamore, 2013). Intriguingly, a recent study has showed that plant-derived exosome miRNAs interact with gut microbiota and indirectly maintain gut health. MiR7267-3p binds to the “TGGCTGGA” region of ycnE mRNA which encoding Lactobacillus rhamnosus monooxygenase, and resulted to increased indole-3-carboxaldehyde level. Subsequently, aryl hydrocarbon receptor-mediated pathway was activated, leading to the expression of IL-22 and evoking antimicrobial immunity and tissue repair responses in mice (Teng et al., 2018).

Among plant-derived miRNAs, medicinal herb derived-miRNAs have drawn a lot of attention because these active ingredients can be absorbed through diets, and most people in developing countries are still rely upon the herb medicine for the treatment of diseases. A large-scale analysis has discovered thousands of small RNAs derived from 10 TCM herbs and most of these miRNAs can be delivered to human blood cells and tissues after oral administration of herbal decoctions (Huang et al., 2019). One question is to what extent herb miRNAs contribute to the known activities of individual herb? Compared with miRNA alone, administration miRNA together with plant extracts showed higher stability and delayed kinetics curve (Zhang et al., 2019). Notably, studies have indicated that therapeutic effects of herbal medicines may attributed to both the chemical compounds and miRNAs in herb (Hamed et al., 2018). For instance, Pastrello et al. (2016) compared the anticancer effects of the main compounds and miRNA in broccoli. It turned out that among the tested 11 genes, 6 were down-regulated solely by miR160, one down-regulated by both miR160 and sulforaphane, and one by miR160 and the active compound in broccoli (3,3-diindolylmethane). Therefore, the interaction between chemical compounds and miRNAs in plant should be considered in future clinical application.

Since the study of cross-kingdom regulation of exogenous plant miRNAs is currently at an early stage, controversy exists regarding the detection and effectiveness of consumed plant miRNA, as well as the repeatability of the published results. In some studies, plant miRNAs were not detected or the levels of target miRNAs were no higher than the background (Mico et al., 2016). Generally, the basis for clinical use of exogenous miRNAs is their exceptional stability toward harsh gastrointestinal environment. Due to the various sequence, structure and binding lipoproteins, miRNAs exert different stabilities, which may fundamentally influence their *in vivo* activity (Zhang et al., 2019). For example, unlike other plant miRNAs, MIR2911 from honeysuckle decoction is resistant to RNase treatment or even boiling process, because of its unique sequence and high GC content (Zhou et al., 2015). Intriguingly, sequence variance not only affects the stability of plant miRNA, but also determines its bioactivity. Recently, study of silencing efficacy of plant variants of miR168a on human LDLRAP1 has demonstrated that although predicted energy scores and miRNA-mRNA duplex structures are similar among rice, tomato and apple miR168a variants, a single nucleotide difference at position 14 lead to the formation of G:U wobble in tomato and apple miRNA-mRNA duplex, results to the significantly decreased LDLRAP1 expression-regulation activity of tomato and apple miR168a variants (Lang et al., 2019). Tripathi et al. (2018) have also found that miRNAs from two tomato varieties showed completely different response to tomato leaf curl virus infections. This reminds us that to ensure the repeatability of previously reported work, plant materials and experimental conditions should be strictly followed. Moreover, it is demonstrated that the concentration of miRNA should higher than 100 copies per cell to effectively suppress the target-containing transcripts (Brown et al., 2006). Therefore, ingestion of insufficient concentration of miRNA, poor bioavailability (Huang et al., 2018), and degradation could all lead to the reduced effectiveness of plant-derived miRNAs. Lastly, improper handling, incorrect selection of endogenous references and contamination of dietary miRNAs may also lead to false results.

Taken together, further efforts should be devoted to two aspects, one is to discovery more novel plant miRNAs and validate their function in human disease treatment, because many bioinformatic predicted miRNA targets were invalid when it comes to *in vivo*. The other is to elucidate the mechanisms and any potential risk of these new active therapeutic substances. The versatile cross-kingdom regulation of plant-derived miRNAs summarized in present study, may lay the foundation for further investigation and application of these newly recognized, non-toxic, and inexpensive plant active ingredients.

## Author Contributions

DL, JY, and YY did the investigation, resources, and data curation. DL, JL, and HL did the writing and draft preparation. RL, CC, and KH revised and edited. LS did the visualization. WW and KH did the supervision and discussion. All authors have read and agreed to the published version of the manuscript.

## Conflict of Interest

The authors declare that the research was conducted in the absence of any commercial or financial relationships that could be construed as a potential conflict of interest.

## Abbreviations

BPH: Benign prostatic hyperplasia
DCs: Dendritic cells
EAE: Experimental autoimmune encephalomyelitis
GE: *Gastrodia elata*
HD: Honeysuckle decoction
JAK-STAT: Janus kinase-signal transducer and activator of transcription
JAMA: Junctional adhesion molecule A
LDLRAP1: Low-density lipoprotein receptor adapter protein 1
miRNAs: MicroRNAs
PBMC: Peripheral blood mononuclear cells
TCF7: Transcription factor 7
TCM: Traditional Chinese Medicine.
